# Electric-Field-Induced Metal-Insulator Transition for Low-Power and Ultrafast Nanoelectronics

**DOI:** 10.3390/nano15080589

**Published:** 2025-04-11

**Authors:** Mircea Dragoman, Daniela Dragoman, Mircea Modreanu, Silviu Vulpe, Cosmin Romanitan, Martino Aldrigo, Adrian Dinescu

**Affiliations:** 1 National Institute for Research and Development in Microtechnologies, Str. Erou Iancu Nicolae 126A, 077190 Voluntari, Romania; vulpesilviu@psg.unibuc.ro (S.V.); cosmin.romanitan@imt.ro (C.R.); martino.aldrigo@imt.ro (M.A.); adrian.dinescu@imt.ro (A.D.); 2 Physics Faculty, University of Bucharest, PO Box MG-11, 077125 Bucharest, Romania; danieladragoman@yahoo.com; 3 Academy of Romanian Scientists, Str. Ilfov 3, 050044 Bucharest, Romania; 4 Tyndall National Institute, University College Cork, Lee Maltings, Dyke Parade, T12 R5CP Cork, Ireland; mircea.modreanu@tyndall.ie

**Keywords:** Mott materials, ferroelectrics, transistors

## Abstract

We present here a comprehensive review of various classes of electric-field-induced reversible Mott metal-insulator materials, which have many applications in ultrafast switches, reconfigurable high-frequency devices up to THz, and photonics. Various types of Mott transistors are analyzed, and their applications are discussed. This paper introduces new materials that demonstrate the Mott transition at very low DC voltage levels, induced by an external electric field. The final section of the paper examines ferroelectric Mott transistors and these innovative ferroelectric Mott materials.

## 1. Introduction

The metal-insulator transition (MIT) physical phenomenon occurs in many solid-state materials, and it entails a dramatic change in the electronic band structure within the considered material; i.e., the electronic band structure possessing an energy gap, specific for a semiconductor or an insulator, is transformed into a gapless electronic band structure specific for a metal, under the action of different stimuli, such as pressure, temperature, and an external electric field. This transformation is, in general, reversible. The acronym MIT is denoted in many papers as IMT, which carries the exact same meaning, i.e., the reversible transformation between a metallic and an insulating state within the same material produced by various external parameters. This transition is accompanied by a significant change in the physical properties of the material, expressed by an abrupt change in the current versus voltage dependence at a certain value of the applied external parameters, since the electronic band structure of the material changes very rapidly from an insulator (in which the current is very low) to a metal (in which the current is very high).

There are three distinct physical mechanisms driving the MIT [1]. The first type is the Mott MIT, due to electron–electron interactions or correlations [2,3]. There is an entire family of materials termed as Mott materials [4], the most well known being vanadium oxide (VO_2_). The band structure of solid-state materials shows that the Fermi level $E_{F}$ is located in the bandgap for the insulators and in the conduction bands for metals, meaning that the metallic behavior is attributed to various materials with partially filled bands. In deep contrast, the Mott materials have partially filled bands but are insulators and not metals—unsuccessful metals, as termed in [4], thus contradicting the band theory.

The reason for such a behavior could be explained as follows: Mott materials contain a large number of carriers interacting via Coulomb forces and, as a result of these interactions, a bandgap is opened. These Coulomb interactions localize the electrons, while interactions induced by external parameters such as temperature, pressure, DC electric field, and electromagnetic field, delocalize the electrons. Hence, the Mott MIT is a reversible localization–delocalization process of electrons changing the electronic band structure of the material under testing from an insulator (localized electrons) to a metal (delocalized electrons). Typical examples of materials exhibiting the Mott MIT are vanadium oxide and nickel oxide, and many other materials termed as transition metal oxides (TMOs).

The second type of MIT is attributed to Anderson, where the localization is produced by disorder. The materials, which are heavily doped ones, such as heavily doped semiconductors, are considered extremely conductive, so that their metallic behavior can be transformed into an insulating one, because a high density of defects could lead to the localization of electrons. This localization has a quantum effect due to the multiple scattering paths making electron waves interfere: this strong interference freezes electron waves inside a medium, and thus, the localization is produced [5]. Hence, the Anderson localization or Anderson insulator is due to quantum interference. Anderson localization is a wave phenomenon encountered in light localization [6], in Bose–Einstein condensates, and in many other physical systems.

The last MIT is named Peiperl’s MIT and is due to electron–phonon interactions. If this interaction is strong, the atoms in the lattice start to oscillate and the symmetry of the lattice is broken.

**Figure 1:**
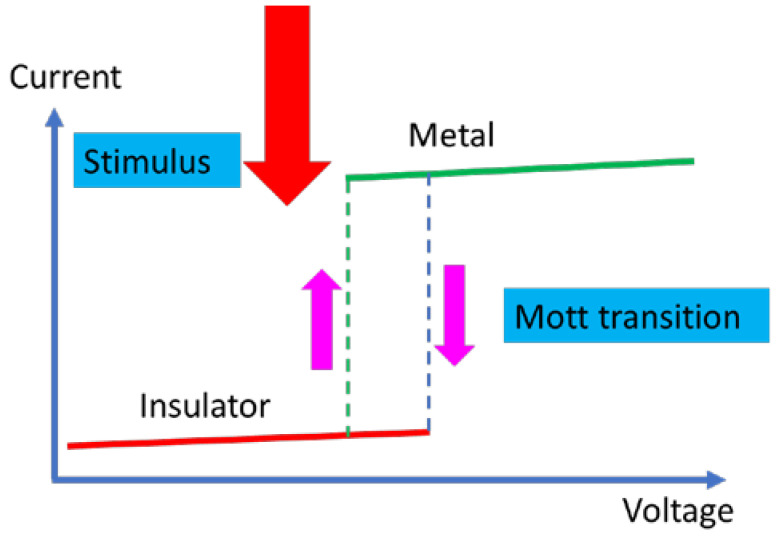
The signature of Mott MIT in the current–voltage dependence irrespective of the applied stimulus.

In this review, we will focus on the electric field able to induce a Mott MIT in various materials, since an electric field is the single external stimulus (among many others) that can be used in nanoelectronics. The physics of the Mott MIT is extensively described in the above references [1,2,3,4,5,6]. Why are Mott materials searched? First, it is because the Mott MIT is very fast and very sharp, and thus, any Mott material is an ideal ultrafast switch with applications in microwaves, as well as in transistors with subthreshold swing below 60 mV/decade (as in the case of CMOS transistors), which are suitable in low-power logic applications. The MIT materials can be applied also in generation/detection of THz signals due to their nonlinear behavior. Second, since the Mott MIT is reversible, it can form an ultrafast resistive memory. All these properties are depicted in Figure 1.

We see in the figure that the sharp transition due to the Mott MIT takes place at a certain critical value of the external stimulus. Although we are focused here on the electric field as a stimulus, other stimuli such as temperature, optical waves, pressure, and strain trigger the Mott MIT as well. For example, the Mott MIT in VO_2_ takes place at 68 °C, which is a rather low temperature, easily achievable in any circuit, so that VO_2_ is extensively used in MIT applications. However, it is much faster to induce the Mott MIT using an applied DC voltage.

The requirements for any electric-field-induced Mott MIT must be (i) ultrafast transition from the insulator state to the metal state, (ii) reversibility, and (iii) repeatability.

## 2. Nanoelectronics Devices Based on Electrically Driven MIT

### 2.1. Ultrafast Nanoelectronics Switches Based on Electrically Driven MIT

An electrical switch is a device having two distinct current states, denoted by ON and OFF. Between these states, the current ratio should be of at least three orders of magnitude. The most used electrical switches are based on semiconductors, where the conduction of carriers is enabled (ON state) or disabled (OFF state) via a control parameter, which is the applied DC voltage in the case of two-terminal devices (such as diodes) or a gate voltage in the case of three-terminal devices (such as transistors). Digital electronics is based on these simple principles, and a microprocessor contains billions of transistors integrated on a single chip representing billions of electronic switches dedicated to computing and memorizing a huge quantity of information in a very short time. These digital switches have nanosized dimensions, their ultimate size being determined by their implementation with atomic-thin materials, which open the path toward electronics at the atomic scale [7]. The transistors used as switches have experienced a progressive reduction in their size and are now crammed into single silicon (Si) chips, numbering a couple of billion in actual microprocessors [8]. This huge progress in transistor integration into a single chip and the continuous improvement of their performance is the result of progress made in almost all areas of applied sciences, in particular physics and chemistry.

Today, transistors based on various semiconductors work up to THz frequencies [9]. This means that a transistor can switch in 1 ps or even less. However, developing fabrication technology and semiconductor materials for ultrafast transistors, such as indium phosphide (InP), is very challenging. Consequently, there is an ongoing effort to enhance the speed of electrical switches for applications demanding ultrafast devices, including quantum measurements, spectroscopy, imaging and sensing, biology, and high-data-rate communications [10].

**Figure 2:**
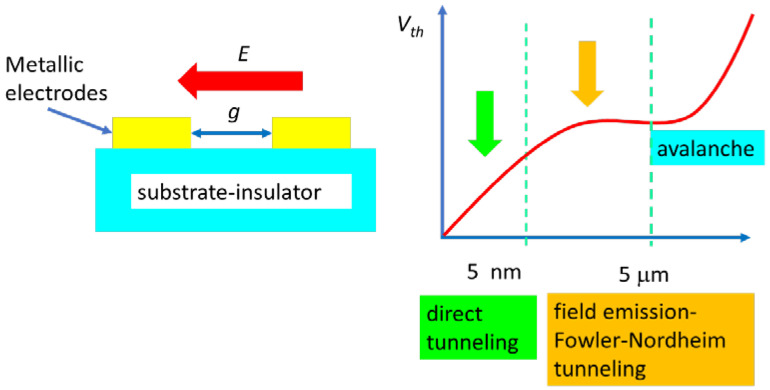
Nanoplasma ultrafast switch.

In this respect, several physical principles are used. For instance, nanoplasma produced in a nanogap, i.e., a nanosized gap between two metallic electrodes, enables the generation of electrical pulses higher than 10 volts per picosecond, which is about two orders of magnitude larger than that of field-effect transistors and more than ten times faster than that of conventional electronic switches [11]. This nanoplasma is formed in the air gap by electrons in two ways: (1) direct tunneling, when the gap size is a few nm and the air gap is modeled as a rectangular barrier, and (2) when the gap is larger, we deal with Fowler–Norheim tunneling or field emission; i.e., electrons cross a triangular barrier formed between the electrodes in atmospheric air when a higher electric field is applied and the barrier becomes triangular (Figure 2).

An electronic device consisting of a metallic tip-to-edge nanostructure with asymmetric sub-10 nm air channel generated an ultrafast response in the sub-picosecond range at a low turn-on voltage around 0.7 V [12]. However, the best results were obtained only at liquid helium (He) temperatures.

Initially, the nanoplasma switch is in an OFF state, since air separates the two electrodes. We need a certain threshold electric field $E_{th}$ to drive the carriers between electrodes. If the nanogap width is less than 5 nm, we are dealing with direct tunneling carriers between electrodes, while if the width is higher than 5 nm and up to 5 μm, the electrons travel from one electrode to the other and tunnel the air gap via field emission or the Fowler–Norheim tunneling process. The electric field required for direct tunneling (through a rectangular barrier) through air is low, whereas Fowler–Norheim tunneling occurs at higher electric fields, for which the rectangular barrier becomes triangular. The physical principles behind these ultrafast devices based on space-charge effects, the tunneling in a nanosized gap and the associated ballistic transport, are described in several review papers (see [13,14]).

Hence, when we apply an electric field in a nanogap located in air we are dealing with an exponential increase in the conductivity due to tunneling. However, this is not a MIT, since in the case of the Mott MIT, the electronic band structure of the material is entirely changed from that of an insulator into that of a conductor. The problem is that the applied electric field is around 4–5 MV/cm, meaning that serious endurance issues can occur, as in the case of nanoscale ferroelectrics with a coercive electric field of a few MV/cm. Such high electric fields can easily damage the metallic electrodes, making endurance and reproducibility persistent challenges that remain to be addressed.

**Figure 3:**
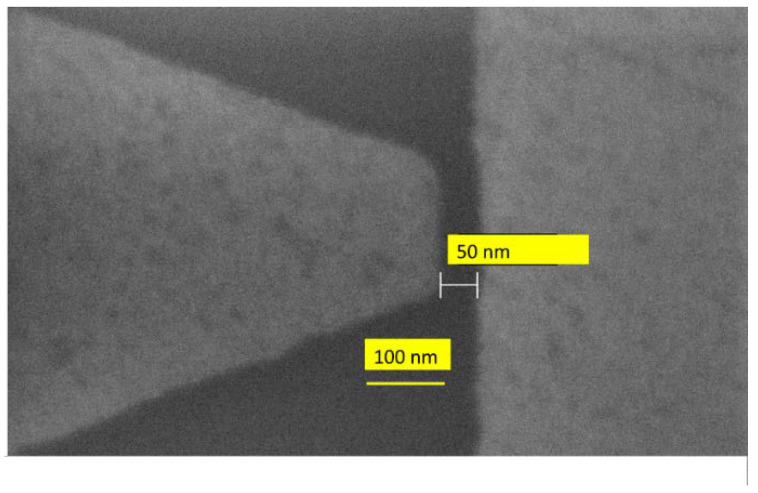
SEM image of a graphene monolayer nanogap (reproduced from [17]).

The above results are inspired by the studies of lateral tunneling in a metal–insulator–metal (MIM) diode, where the dielectric is air. In this way, this lateral tunneling graphene diode can be elevated as a member of the nanoscale air channel devices [15]. The electric field is significantly enhanced at the sharp tip of graphene and thus, the tunneling efficiency is increased by reducing the barrier height/increasing the electric field in the region with a high radius of curvature at the graphene tip. Since the gap width is 20 to 30 nm in [16], the tunneling time is very short, i.e., around 10–20 fs, which translates into a cutoff frequency of tens of THz. These results make lateral-tunneling MIM diodes the fastest electronic devices, since we have detected with such graphene nanogap MIM diodes IR signals having a wavelength of 2 μm [17] (Figure 3).

There also exist ultrafast switches related to photonic technologies, where MIT plays a key role. The reader could find some excellent reviews related to these issues [18], with optical switches working even in the attoseconds range [19].

In deep contrast with the above-mentioned ultrafast switches, the Mott MIT ultrafast switch is a device where the material itself switches very fast from an insulator state to a metallic state in a controlled and reversible way. A very good review of Mott materials and their physics is found in [20], and it will not be repeated here. The ultrafast switch is thus an intrinsic property of the material, and the switching time depends on the Mott material growth technique and the natural stimulus to produce an MIT. While voltage pulses produce MITs with a switch speed of a few ns up to fractions of ns, the optical excitation produces switching speeds in the range of fs (see Table 2 from [21]).

**Figure 4:**
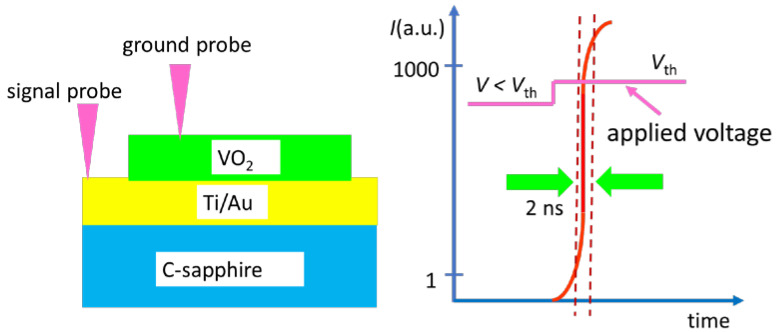
The ultrafast pulse MIT in VO_2_ in a MIM configuration. The current increases abruptly when the applied voltage reaches a threshold value *V*th.

Let us examine in detail this ultrafast response Mott material when applying electrical pulses to various types of vanadium oxides. We start with the most used material, i.e., VO_2_, excited by voltage pulses. There are recent review papers where different kinds of vanadium oxides and their applications are presented [22,23]. It is shown that VO_2_ grown by magnetron sputtering at 550 °C on Ti (20 nm)/Au (200 nm) with a thickness of 400 nm and deposited on a *c*-sapphire in a MIM structure configuration (Figure 4) is able to switch in 2 ns from a state with a resistance 2.4 × 10^4^ Ω (insulator) to a state with 150 Ω (metallic) [24], when a 2.5 μs electrical pulse is applied having an amplitude of 2.5 V. A similar ON/OFF ratio in resistance/current, i.e., 10^3^–10^4^, is obtained when the MIT is induced thermally. There are other types of vanadium oxide that demonstrate the same ultrafast response, with a switching time less than 1 ns when voltage pulses are applied [25].

The physical origin of the ultrafast switch response induced by applied electric fields is under debate in relation to the fact that the current induced in VO_2_ produces heating of the material; hence, the MIT could be produced by the Joule effect and/or have a non-thermal origin.

**Figure 5:**
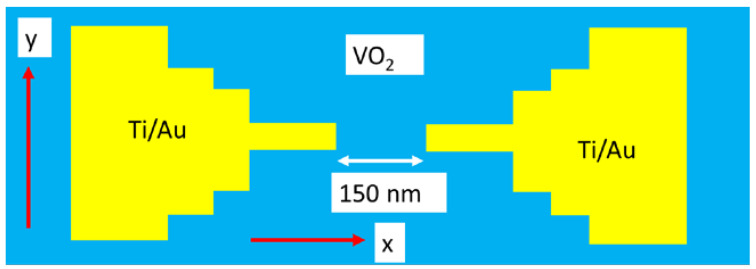
The schematic of an MIT-based VO_2_ thin film, with a typical length of 150 nm and width of 100 nm.

The electrically driven MIT by Joule effect generating an ultrafast response is explained by a model developed in [26]. According to this model, the pure Joule heating inducing an ultrafast MIT is based on narrow ribbons of VO_2_ (widths of 100 nm) and narrow gap electrical contacts (Figure 5).

Based on the experimental results in VO_2_ thin films with a thickness of 200 nm, consisting of excitations with pulses with a rise time of 5 ns, a theoretical two-dimensional model was developed based on the following Fourier equation:

(1) $${\rho_{0}C}_{th}\partial T/\partial t=k[(\partial^{2}T/\partial x^{2})+(\partial^{2}T/\partial y^{2})]+J_{y}^{2}\rho_{r}-H_{eff}(T-T_{0})/A$$

where $\rho_{0}$ is the material density, $C_{th}$ is the specific heat capacity, T is the temperature, k  the thermal conductivity, $J_{y\;}$ is the current density in the y direction, $\rho_{r}$ is the resistivity, $H_{eff}$ is the effective surface heat transfer coefficient per unit length, and A is the area.

**Figure 6:**
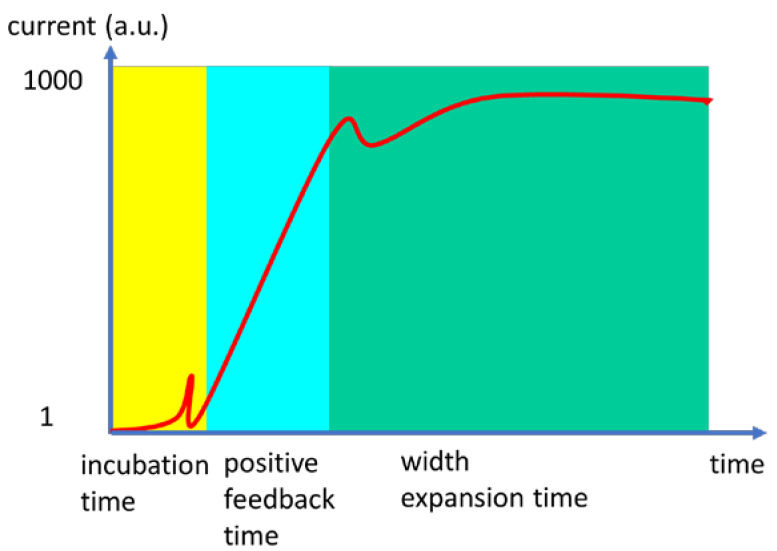
The ultrafast response steps of an MIT material.

According to the above model and in agreement with pulse measurements, it was observed that the formation of ultrafast pulses occurs in several steps (Figure 6). The first step refers to the incubation time, when the current is low, the second step consists of a positive feedback time, when the current increases abruptly, and the final step, when the current saturates, is termed width expansion time.

Up to the incubation time $t_{inc}$, the temperature is increasing due to the Joule effect but is below the critical temperature at which the MIT is produced (in VO_2_, at 65–68 °C depending on the growth method). In this regime, the current is low, since the material is in the insulator state. The incubation time thus represents the delay of the MIT device until when the external stimulus produces the MIT. By denoting $V_{exc}$ as the amplitude of the applied electric pulse and $V_{C}$ as the critical voltage value when the MIT occurs, we have

(2) $$t_{inc}={const\times (V_{ex}-V_{C})}^{-0.5}$$

This means that a low incubation time is found when the amplitude of the pulse is high enough to rapidly heat the material.

The positive feedback time region corresponds to the regime when the MIT takes place, and hence, the current increases with orders of magnitude; this region is relevant for the ultrafast time response of the MIT.

During the width expansion time, several oscillations occur due to the spread of Joule heating filaments in the material. A non-thermal MIT is also possible, which could be faster than a thermal MIT. However, it is difficult to distinguish between thermal and non-thermal effects, since very often they are correlated. There are different approaches to separate these two effects: (i) use very small devices, as indicated above, where a narrow gap (70–150 nm) and a small material width fit with the current filamentary widths; (ii) grow an epitaxial film to obtain a uniform single-crystal; (iii) perform measurements at low temperatures in order to reduce leakage currents and thus heating. All these requirements are satisfied by V_2_O_3_ thin films grown by molecular beam epitaxy on a *c*-sapphire substrate with a thickness of 50 nm [27]. In these films, it was found that the incubation time depends on the amplitude of the applied electrical pulse and the temperature. The short incubation time, due to purely thermal effects, was 150 ps at 100 K.

**Figure 7:**
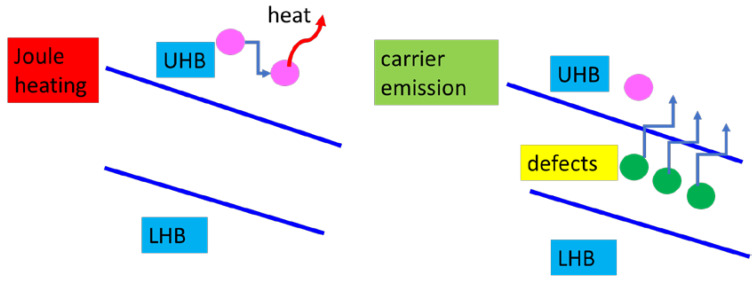
The schematic diagram for the thermally and non-thermally induced MIT.

However, non-thermal resistive switching of the Mott MIT is preferred due to its significantly lower energy consumption compared to Joule heating. This allows for devices based on it to play a crucial role in various applications such as memories and neuromorphic computing. A non-thermal Mott MIT was achieved by doping VO_2_ and V_2_O_3_ nanowires [28] via irradiation with Ga ion beams in order to create controllable defects in the nanowires. In the case of lower doses, the thermal MIT is observed, while at high doses the nanowires are driven to Mott MIT by field-assisted carrier generation, in which case the MIT is determined by doping (Figure 7). The nanowires, with a width of 100 nm and a length of few micrometers were grown using magnetron spattering. The width of these nanowires match that of the metal-insulator domains, suppressing thus the filament formation during MIT, and therefore slowing down the spread of the heat in the entire structure. More precisely, the physical phenomena taking place can be understood looking at the electric field tilting of the Hubbard upper band (UHB) and the Hubbard lower band of Mott insulators. In a defect-free Mott insulator, only a small number of activated carriers are within the UHB and, when a high electric field is applied, these carriers are scattered, generating Joule heating. On the contrary, in a doped Mott insulator, the defects, which are in-gap states, are transported in the UHB by a moderate electric field and the MIT is produced by doping due to the Poole–Frenkel effect (Figure 7).

The thermal-induced MIT remains today the main research route for applications of Mott materials at high frequencies. In particular, the thermally induced MIT in VO_2_ was used extensively in the microwave and millimeter-wave region to fabricate and test radiofrequency (RF) switches and reconfigurable antennas based on them, as well as in filters, sensors, and metasurfaces, which were tested and yielded good results [29].

Terahertz (THz) and optically induced MIT can produce ultrafast responses in the range of femtoseconds (from a few to hundreds of femtoseconds) depending on the type of laser excitation, wavelength, and power. All research in this respect is performed on the prototype material VO_2_, where MIT is thermally induced, and has important applications in THz devices [30,31,32,33]. Reconfigurable antennas, metasurfaces, absorbers, and resonators are envisaged.

**Figure 8:**
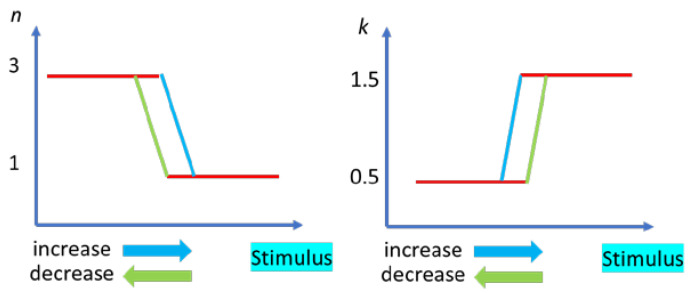
The schematic behavior of the index of refraction, *n*, and extinction coefficient, *k*, during MIT induced by any of stimulus, including an applied electric field.

Other important applications of MIT materials are in nanophononics. In the case of optical applications, the reflection and transmission of electromagnetic waves are monitored when a stimulus (electric or thermal) is applied on the MIT material. In this case, the variation in the refractive index is the key parameter for tunable optical devices, such as modulators, metasurfaces and metamaterials, or plasmonic antennas, spatially addressable for optical memories [34]. The variation in the complex index of refraction $n_{opt}=n+ik\;$ in an MIT material has the same hysteretic shape as the conductivity of the MIT and is dependent on the wavelength and the strength of the stimulus. Figure 8 schematically represents the behavior of the real and imaginary parts of the complex index of refraction of an MIT material.

This figure illustrates the fact that, for a critical value of the stimulus (either temperature, voltage or pressure), the index of refraction decreases from a value typical for an insulator towards that of a lossy metal. On the contrary, the absorption is initially low, but it increases beyond the critical value, expressing a lossy metal. When the MIT material is VO_2_ and the stimulus is the temperature, the changes during the MIT of the refractive index, ∆n, is 1.93, and that of the absorption/extinction, ∆k, is 0.77 at a wavelength of 1 μm [35].

**Figure 9:**
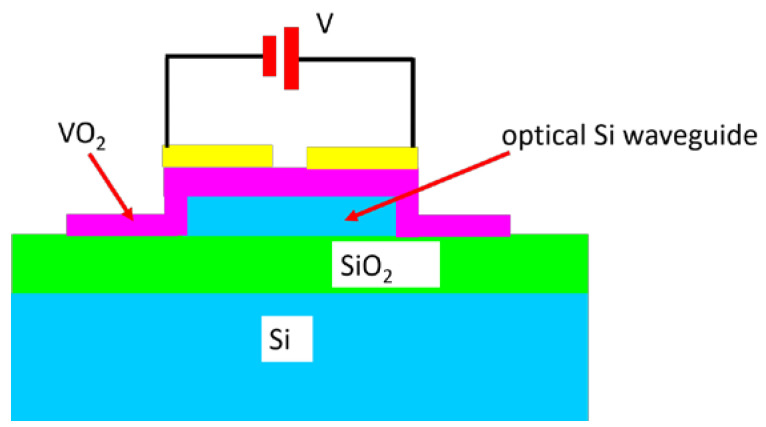
Schematic cross-section of the electro-optic modulator based on voltage-driven MIT in VO_2_.

Although the state of the art is based on optical devices that are thermally driven to MIT, some important alternative results were reported in the case of VO_2_. For instance, an absorption electro-optical modulator based on electrically driven MIT in VO_2_ [36], presented in Figure 9, was developed.

In fact, this electro-optic modulator is able to reveal the electrical switching of VO_2_ using optical methods. The DC bias applied on a 100 nm gap configuration is used to create a high electric field, of the order of 10^7^ V/m, which is necessary for MIT and minimizes the leakage currents. The optical signals propagating in the waveguide are affected by MIT, while the low-power optical signal propagation does not perturb the phase of VO_2_, which is controlled by the voltage V applied between the gold electrodes. It is found that MIT is induced at *V* = 1.7 V, corresponding to 2 × 10^7^ V/m within the VO_2_ film with a thickness of 60 nm, and exhibits an abrupt current jump of one order of magnitude. Time domain and temperature measurements are also performed. The MIT is faster than the resolution of the measurements limited by a 2 ns rising time of the input voltage pulse. The transmission in the single-mode optical waveguide depends strongly on the applied voltage, since the evanescent modes of the waveguide extend evanescently in VO_2_. When no DC voltage is applied, the modulator is in an ON state (0 dB), whereas the transition to a metallic state increases the absorption when MIT is produced, so that the modulator is driven in the OFF state (−8 dB at a wavelength of 1500 nm).

**Figure 10:**
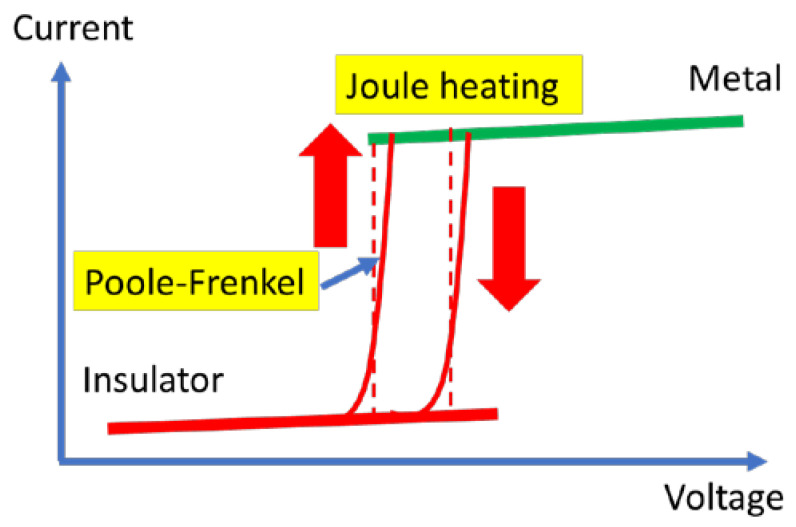
The electric field drives MIT physical mechanism.

Experiments and simulations have shown that there are two time scales of the electrical pulse-driven MIT. The first, short-scale process is due to a high electric field coming from the injection of electrons in the insulator (often termed semiconductor) VO_2_ via the Poole–Frenkel effect, producing rapid heating in less than 1 ns. The Poole–Frenkel effect describes the conduction of electrons in an insulator in the presence of a high electric field. The Poole–Frenkel effect is similar to the Schottky effect associated with the lowering of the metal-insulator energy barrier, and the current density dependence on the applied voltage is given by

(3) $$J=V\exp\left[(e/k_{B}T)[2{(eV/4\pi \varepsilon t)}^{0.5}-\emptyset_{B}\right]$$

where $k_{B}$ is the Boltzmann constant, ε is the dielectric permittivity, t is the dielectric thickness, and $\emptyset_{B}$ is the voltage barrier (in the absence of an applied electric field). Equation (3) models the abrupt, exponential increase in the current during MIT, which is presented in Figure 10.

**Figure 11:**
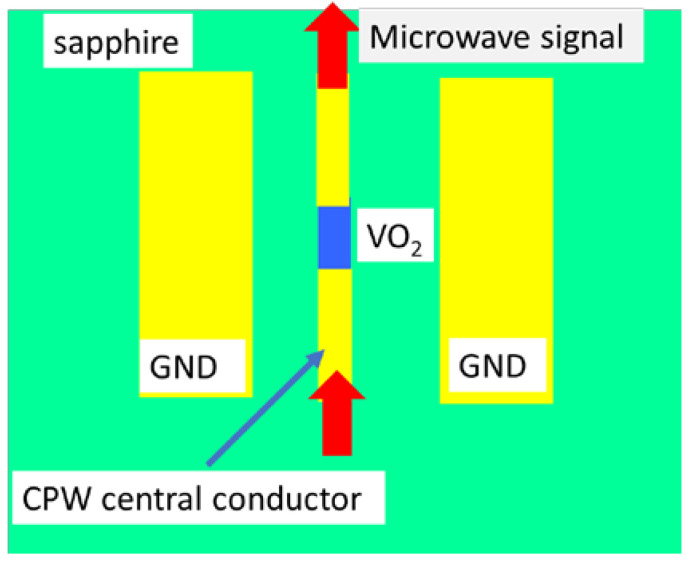
Microwave coplanar waveguide MIT device using VO_2_ thin films.

Then, as the current flowing between the metallic gaps separated by 100 nm in the VO_2_ device described increases, the Joule heating is spreading the current filament with a rate of about 20 nm/ns in the whole VO_2_ area. This is a longer time process, depending on the area of the Mott material and the duration of the excitation electrical pulse, which should be the shortest possible in order to reduce the time of the heating process, which is no longer localized in the gap between the electrodes. Similar two-time scales are observed when the optical signals are replaced with microwave signals [37] in a microwave-guided structure termed as a coplanar waveguide (CPW), which consists of three metallic electrodes deposited on Al_2_O_3_, i.e., a sapphire crystal (0001) substrate, where the central conductor is the microwave signal electrode and the other two are ground electrodes. The VO_2_ is grown by RF magnetron sputtering with a thickness of 120 nm, etched at the dimensions of *L* = 50 μm and *W* = 25 μm, and placed in the gap of the central conductor as indicated in Figure 11.

The above device allows measurements of thermally or electrically driven MIT in VO_2_ such that a comparison between these two effects could be made. The change in the resistance, defined as $∆R=R_{OFF}/R_{ON\;}$, is denoted as ${∆R}_{E}$ when it originates from electrically driven MIT experiments, and by ${∆R}_{T}$ for thermally driven MIT experiments. In many cases, ${∆R}_{E}$ < ${∆R}_{T}$, but in the device in Figure 11, it was found that ${∆R}_{E}$ ≈ ${∆R}_{T}$ = 10^3^. The explanation of this result resides in the fact that in the case of thermally driven MIT, the entire area of VO_2_ undergoes a Mott transition, while when a voltage is applied, the electric-field-driven MIT does not occur across the entire VO_2_ area due to non-uniformities in the film composition, defects, or crystallographic orientation, briefly due to the growth method employed for VO_2_ thin films. The time-domain analysis has shown that there are, again, two scales: an abrupt increase/decrease in conductivity having a rising/falling time of the order of 10 ns, followed by a slow, oscillatory time that takes a few μs. This is an RF switch up to 14 GHz with a flat insertion loss of 3 dB and an isolation of at least 25 dB, when the switch is biased at 10 V and the currents have values between 70 and 80 mA.

The examples above demonstrate that the electrically driven MIT of VO_2_ has become a solid path toward tunable devices working in microwaves or photonics, such as metasurfaces [38], modulators [39], and optical memories [40].

### 2.2. Nanoelectronics Devices Based on Electrically Driven MIT for Low-Power Applications and Computing

Given that our paper focuses on the applications of MIT in nanoelectronic devices, we will discuss further the Mott transistor and its applications. This transistor is the most significant illustration of an electrically driven MIT, controlled by an electrostatic field generated by a DC polarized gate, and is crucial for low-power applications, memories, and computing.

**Figure 12:**
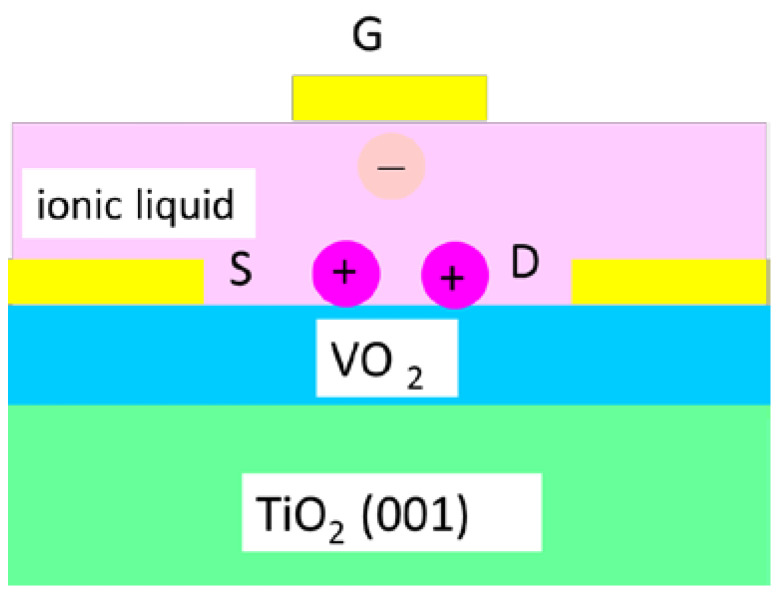
The schematic diagram of an electric-double-layer transistor (EDLT) Mott FET.

The Mott transistor was proposed as an analog of a field-effect transistor (FET), where the current flow between drain and source is modulated by an electrostatic electric field leading to low conductivity and high conductivity values, which correspond to the ON and OFF states of the transistor, i.e., the most effective electrical switch to date with a switching energy between the two states of just a few attojoules [41]. Since the Mott MIT is an ultrafast switch, the idea was to replace the conductive channel of a FET with a Mott material to obtain an ultrafast switch and an ultrafast non-volatile memory. This is the Mott FET [42], but there are difficulties with VO_2_ growth at high temperatures and the control of its physical properties, which depend on the thickness and the chosen substrate. Mott FETs based on VO_2_ with a thickness in the nanometer range will have a switching time of 0.5 ps and a transfer power of 0.1 μW [43]. So, different solutions were found to build a Mott FET. For instance, a VO_2_ electric-double-layer transistor (EDLT) was developed, which is a Mott FET with an ionic liquid gate. The electrostatic charging at the VO_2_ surface delocalizes the charges in the bulk, creating a fully metallic state (Figure 12).

The electrochemical cell starts working when a gate voltage is applied and, as a result, the ions move to the gate and the 10 nm VO_2_ channel. The cations are electrostatically absorbed by the VO_2_ surface to form a double-layer capacitor having a sub-nanometer gap. In this way, the organic ionic liquid is able to tune the surface charge density up to 10^15^ cm^−3^. At the transition temperature of 260 K of the VO_2_ channel, the sheet resistance is changing reversibly with more than two orders of magnitude when the gate voltage changes from −3 V to 3 V, thus reflecting the reversible transition between the tetragonal metallic phase and the monoclinic insulating phase [44]. However, this is a slow FET due to the ionic liquid, which could also chemically interact with the materials forming the electrochemical cell.

**Figure 13:**
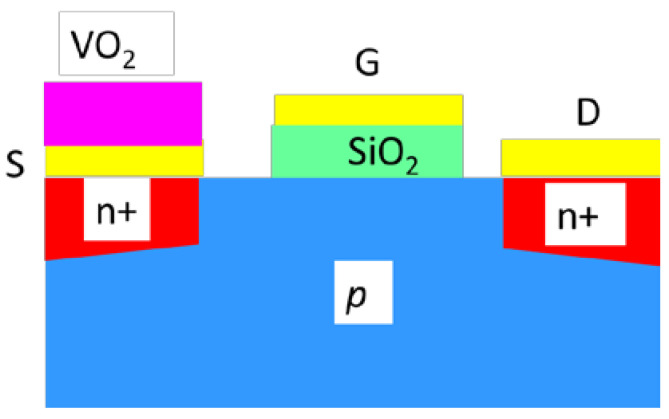
The schematic diagram for VO_2_-based Hyper-FET.

The Hyper-FET was the first demonstration of a Mott FET with an excellent current–voltage dependence showing a subthreshold swing below 60 mV/decade, thus breaking the Boltzmann tyranny. Boltzmann tyranny means that CMOS transistors cannot remove entirely the heat generated during the switching process. This thermal limitation is expressed by the subthreshold swing $SS=\partial V_{G}/\log(I_{D})$, which cannot be lower than 60 mV/decade in the case of CMOS FETs. In the case of Hyper-FETs, the SS is able to attain values lower than even 10 mV/decade [45,46]. The VO_2_ thin film is connected in series with the source of an FET [47] and does not replace its channel (Figure 13); the negative differential resistance of VO_2_ is used to produce internal voltage amplification [48] in order to enhance the performances of the FET.

More specifically, VO_2_ plays here the role of a threshold selector. When the VO_2_ is in its insulating state, the FET channel has a large resistance, and a part of the drain voltage $\left(V_{D}\right)$ and gate-source voltage $(V_{GS}$) is reduced by VO_2_, and thus, the leakage current is reduced. By increasing the gate voltages $\left(V_{G}\right)$, VO_2_ arrives in its metallic state, and the channel resistance decreases and the drain current ($I_{D})$ increases abruptly due to the Mott transition. As a result, the *SS* has low values.

The $I_{D}$ shows an abrupt dependence on $V_{D}$ at various $V_{G}\;$ values and on $V_{G}$ at various $V_{D}$ voltages. The measurements are made at room temperature. There is a threshold gate voltage that drives the FET from the OFF state to the ON state $\left(V_{G,IMT}\right)\;$, and another that drives the MIT from the ON state to the OFF state $\left(V_{G,IMIT}\right)$. The difference between these gate voltages gives the hysteresis width, which is about 2 V, while $V_{G,IMT}$ is less than 2 V. The *SS* is below 20 mV/decade at certain values of $I_{D}$. This Hyper-FET architecture can be applied to any transistor configuration, including FinFETs, irrespective of whether it is *n*-type or *p*-type. A record of 8 mV/decade and an increase of 36% in the drain current is obtained in 14 nm node FinFETs [49]. Very good results are obtained for the AlGaN/GaN HEMT, where the source is loaded with a 50 nm VO_2_ grown by ALD on sapphire [50]. The ON/OFF ratio is 10^12^ and the minimum *SS* is 9 mV/decade.

The Hyper-FET and phase-FET, where the MIT material is not an intrinsic part of the FET, were developed because the Mott transition needs a rather high electric field and, thus, many charges that cannot be provided by a dielectric gate. Thus, the modulation of the electric charge carrier density by applying an electric field (as in the case of FETs) is not directly applicable in the case of Mott materials used as channels in a Mott FET, since they are insulators and not semiconductors. The ionic gate FET explained above satisfies this requirement but has several drawbacks, since nanoelectronics today are based on solid-state materials. Another solution to create a large modulation of the carrier density is to apply large electric fields to insulators, creating polarization of the charges [51], i.e., using ferroelectric gating in place of dielectric gating. For example, in Ref. [52], CaMnO_3_ was used as a potential channel Mott material, whereas BiFeO_3_ was deployed as a ferroelectric, thus creating a fourfold channel resistance variation at room temperature and a tenfold resistance variation at 200 K.

Hafnium oxide (HfO_2_)-based ferroelectrics are a good solution, since their relative electrical permittivity is moderately high (20–40), they possess a large band gap (5.5–6 eV) and a large remanent polarization (30–40 μC/cm^2^), in addition to being CMOS compatible, HfO_2_ being currently the gate dielectric used in standard CMOS processes. There is a very large body of work in the literature in the area of HfO_2_ ferroelectricity, obtained by doping it with Zr, Y, S, Ge, and other dopants. Therefore, we mention here just some review papers on the subject [53,54,55]. The motivation of this huge amount of work is related to memories and the low *SS* of ferroelectric FETs—a subject which in common with Mott materials. Thus, the combination of HfO_2_-based ferroelectrics and Mott materials could produce innovative devices for low-power applications. Ultimately, as we will see in the next chapter, there are ferroelectric materials showing Mott MIT, and which work also as very good transistors with very low *SS*.

**Figure 14:**
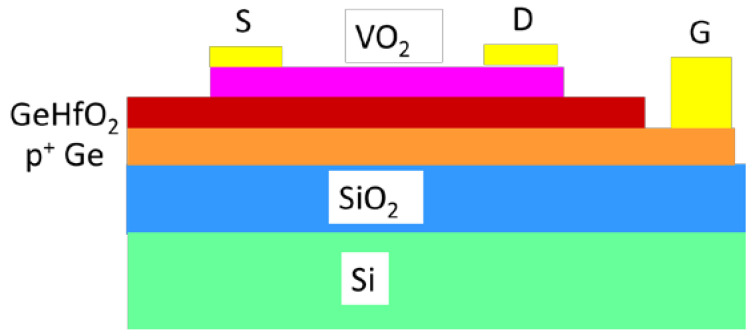
Schematic of a Mott FET based on GeHfO_2_-ferroelectric.

Thus, using the ferroelectric gating, it is possible to fabricate Mott FETs using VO_2_ (thickness of 8 nm) as the channel, grown over the ferroelectric HfO_2_ doped with Ge (thickness of 30 nm) grown in turn on p^+^ Ge (thickness of 39 nm) working as a back-gate, deposited on a Si/SiO_2_ substrate [56] (Figure 14).

The modulation of the conductivity of the VO_2_ channel is possible through ferroelectric polarization switching induced by the applied DC electric field, in combination with the electrically driven MIT. When the polarization is oriented towards the VO_2_ (positive $V_{G}$) the conductivity of the channel is increased due to the increased electron density in the channel. When the polarization is reversed (negative $V_{G}$) the channel is doped by holes, which decreases the conductivity. However, although the electrical switching of the ferroelectric polarization induces MIT, the ratio between the two conductance states is rather low. A modeling of the Mott FET with a VO_2_ channel using a ferroelectric HfO_2_ layer doped with Zr (termed as HfZrO) as gate can be found in [57].

A record room-temperature high resistive switching of about 40% in Mott transistors is obtained having as top gate a PZT ferroelectric and as Mott materials RNiO_3_/La_0.5_Sr_0.33_MnO_3_, where R signifies a rare earth element and La_0.5_Sr_0.33_MnO_3_ is a buffer layer that reduces the depolarization effects and engineers the carrier density profile [58]. However, due to the materials used, such as rare earths (which are critical raw materials) and Pb (which is forbidden in many countries), this transistor is not in the mainstream of FET research, also being incompatible with CMOS processes. Hence, up to now, only the Hyper-FET has demonstrated robust experimental results that could be used further in applications such as memories, logic gates, and neuromorphic computing.

In particular, Mott materials as ultrafast switches for nanoelectronics and photonics are targeting applications in memories and neuromorphic devices. Any Mott material is a resistive switch and a type of non-volatile memory storage, but if we compare them with state-of-the-art memory [59,60] and neuromorphic devices such as memristors, artificial synapses or artificial neurons [61], the respective Mott devices are in their infancy. The main reason is that these materials such as vanadium oxides, rare-earth perovskites, or other Mott materials are difficult to grow and are not integrable with Si technologies, which are the mainstream technology in nanoelectronics. There are recent reviews about Mott memories [21,62] and Mott neuromorphic devices [63,64]; therefore, these issues will not be repeated here.

## 3. New Materials for Electrically Driven MIT

As already mentioned, most applications of Mott materials in electronics, microwave, and photonics are based on VO_2_, which is thermally driven to MIT at 68 °C. However, there are several drawbacks. Any thermally driven device is rather slow and the growth of VO_2_ is not an easy task since vanadium has 11 different oxide phases [65]. Therefore, there are still serious problems regarding stoichiometry, the vanadium concentration in VO_2_ multiphases making the fabrication process dependent on thickness, grain size and distribution, degree of crystallinity, and substrate. As such, experimental results regarding MIT in VO_2_ are very often contradictory, since the control of the fabrication process is not fully accomplished. However, in [65] a fabrication process is established to produce crystalline VO_2_ grown on any substrate using magnetron sputtering, the results being similar for three types of substrates: glass, quartz, and Si. A more detailed review of vanadium oxides phase diagrams, structures, and synthesis is reported in a recent study [23]. Due to the multitude of vanadium oxide types, a lot of research has to be performed still, since only VO_2_ is rather well studied, with other vanadium oxides, such as V_2_O_3_ and V_2_O_5_, being less studied, although they show a thermally driven MIT at 160 K and 530 K, respectively. These efforts are justified by the multiple applications of vanadium oxides in electronics, supercapacitors, batteries, electrochromics, and catalysts.

NiO (nickel oxide) was the first material studied by Mott [66] for MIT, who explained the existence of the energy gap as due to correlated electrons interaction, which opens a bandgap between the Ni 3d UHB (upper Hubard band) and the O2p band. NiO is considered as a wide bandgap semiconductor (3.7–4 eV) and is targeted for CMOS-compatible resistive switching memories (for a recent review, see [67]) and memristors [68]. Besides these applications, NiO is also a ferromagnetic material and a transparent semiconductor, the Ni/NiO/Ni junctions being used as electromagnetic detectors, able to sense THz and infrared electromagnetic signals at room temperature [69]. All these applications were developed without considering the direct connection between the fact that NiO is a semiconductor oxide and a Mott material at the same time, although a couple of results regarding the physical properties retrieved in devices such as resistive switching memories, or the strong nonlinear current–voltage dependence and the ultrafast response of NiO (among the few materials in MIM structures able to detect the infrared radiation) offer at least an indication for MIT. On the other hand, there are many experimental proofs for MIT in NiO with various stimuli, e.g., applied pressure or electric fields. For instance, a colossal resistive switching of six orders of magnitude and a reversible MIT at room temperature at very low applied voltages were shown recently in RNiO_3_ perovskite nickelates, where R is a rare earth lanthanide element, in this case Sm [70]. However, rare earths are critical materials and must be replaced in the near future. Moreover, such perovskite nickelates are unstable over time. Non-stoichiometric NiO shows vacancy-induced MIT at a few volts by electrochemical charging in a Li^+^-containing electrolyte [71]. MIT in NiO is observed by compressing it at high pressures, around 260 GPa [72].

**Figure 15:**
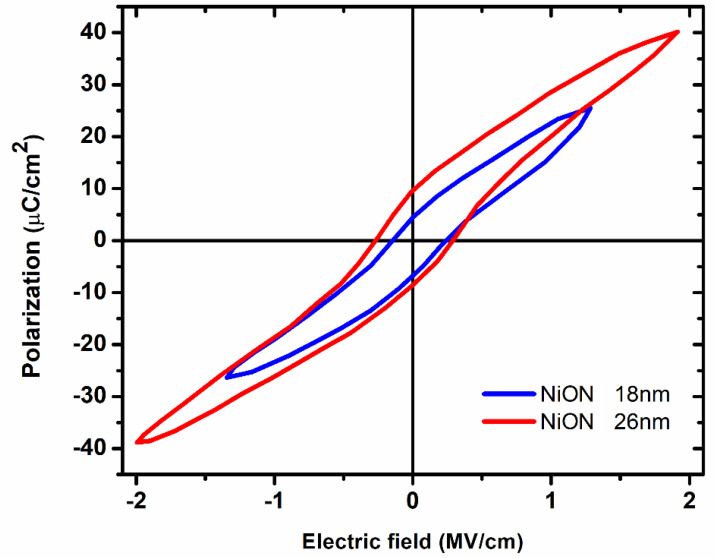
P-E loops for NiON thin films (18 and 26 nm thick) measured at room temperature [73].

Recently, we have shown that NiO doped with N is ferroelectric; more precisely, N-doped NiO (further denoted as NiON) is the first Mott material that is ferroelectric [73] at room temperature. The ferroelectricity in NiON is due to breaking of the crystallographic symmetry by oxygen vacancies created by doping. Specifically, when the N doping level is increased, a transition from a (002) orientation to a (111) preferential orientation of the cubic NiO is observed. This result identifies the distortion of cubic structure in NiO, caused by oxygen vacancies due to doping as the physical origin of ferroelectricity in this material. The finding is confirmed further by extensive material characterization experiments and electrical measurements [73]. Actually, defect engineering is a known, key technique to control the physical properties of materials and to transform these into new crystalline arrangements that are symmetrically forbidden in defect-free materials. The polarization versus electric field dependence in NiON at room temperature is illustrated in Figure 15, which shows that doping breaks the crystallographic symmetry and is able to generate ferroelectricity in centrosymmetric materials by rearrangements of oxygen vacancies. A similar situation occurs when giant piezoelectricity is induced in centrosymmetric oxides by applied electric fields [74]. The same oxygen vacancy defects cause ferroelectricity in HfO_2_ by doping it with Zr, Y, Si, Al, or Ge. As a result, the centrosymmetric HfO_2_ material becomes non-centrosymmetric [55].

Regarding NiON, apart from the P-E loops in Figure 15, more detailed information about the structural characterization (in particular, XRD pole measurements) confirmed the presence of a distorted cubic structure of NiON, while chemical composition studies (XPS and FTIR) confirm the presence of oxygen vacancies in NiON [73]. Note that Raman spectroscopy investigations cannot be performed on NiON films with very small thicknesses, since the Raman response is too small to be measured. More results are found in the newly published paper [75].

Very recently, the first Mott molecular ferroelectric was reported [76] in the inorganic-organic material (C_7_H_14_N)_3_V_12_O_30_. However, the MIT takes place at 234 K, below room temperature, and the remanent polarization at 170 K is very small, of only 2 μC/cm^2^.

**Figure 16:**
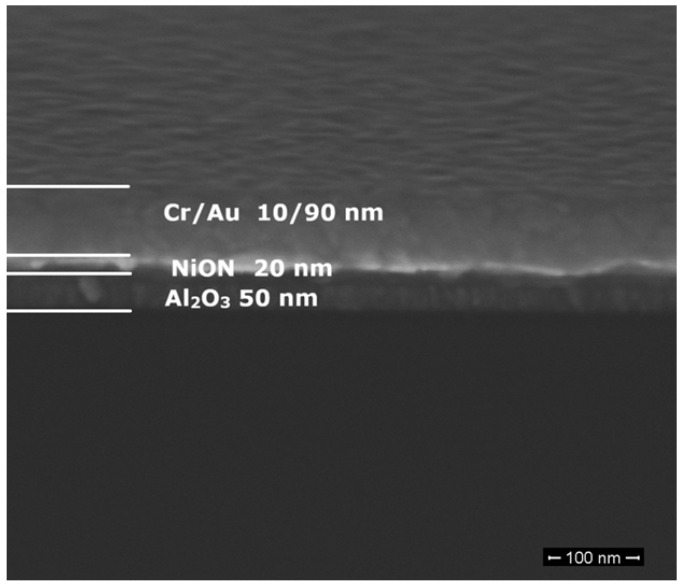
The SEM image of the cross-section of a NiON FET.

Thus, NiON remains a strong candidate for Mott insulator-based devices. We have fabricated tens of FETs based on NiON (thickness of 20 nm) playing the role of the channel grown on aluminum oxide (Al_2_O_3_, thickness of 50 nm) on a 525 µm thick 4-inch wafer of doped Si (playing the role of a back-gate). The NiON channel was deposited from a 6-inch Ni target using the RF sputtering technique in a plasma of Ar-O_2_-N_2_, while the Al_2_O_3_ layer was deposited by atomic layer deposition (ALD). The NiON FETs have a width *W* = 2 μm and a length *L* = 22 μm. The SEM image of the cross-section of such an FET is shown in Figure 16.

**Figure 17:**
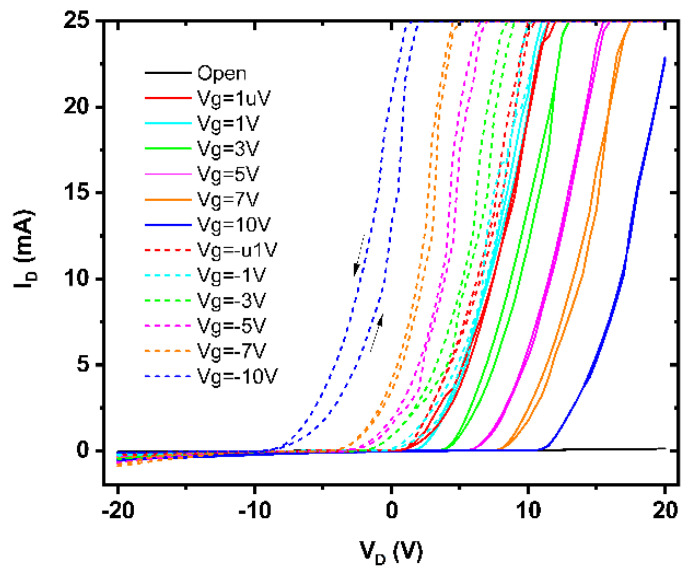
The *I_D_* versus *V_D_* dependences at various gate voltages of a NiON FET.

In Figure 17 we have represented the drain current (*I_D_*) versus drain voltage (*V_D_*) dependences of a NiON FET acting as a sharp switch, when the drain voltage spans the range between −20 and +20 V at very low gate voltage values (*V_g_*), of +1 μV; the drain current switches with five orders of magnitudes and the SS is around 50 mV/decade. As follows from Figure 17, the voltage-driven MIT is tuned by *V_g_.*

Molybdenum trioxide (MoO_3_) was not known previously as a Mott material until recently we discovered its sharp voltage-driven MIT and ferroelectricity. We have recently presented experimental evidence of reversible insulator-metal transition in thin-film amorphous MoO_3_ induced by electric fields of just few volts. The presence of oxygen vacancies in MoO_3_ dictates the reversible MIT. This result is based on numerous GIXRD, UV-VIS-NIR, ellipsometry, XPS, and TEM experiments, as well as on electrical measurements [77].

**Figure 18:**
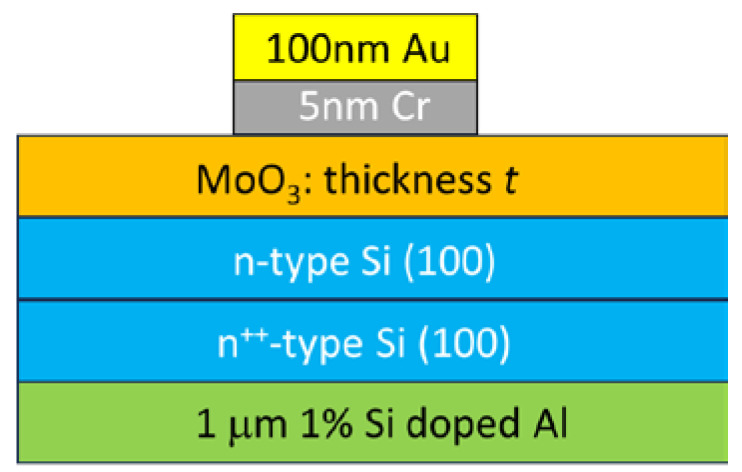
The cross-section of MoO_3_ devices deposited on Si. The MoO_3_ thickness is denoted by *t* [77].

MoO_3_ is a van der Waals semiconductor, having thus a layered structure (a single monolayer is 1.4 nm thick), with a bandgap of 3.2 eV and a stable crystalline polymorph α- MoO_3_, with an orthorhombic crystal lattice. The reported MoO_3_ mobility in FET devices attains 1100 cm^2^/V·s, which is comparable with that of Si [78]. Even an ink of MoO_3-x_ has a mobility of 600 cm^2^/V·s [79]. These mobilities are much higher than in any other 2D semiconductor material and are similar to that of black phosphorus (BP), which is very difficult to grow and preserve [80]. Amorphous MoO_3_ thin films were deposited at room temperature by e-beam evaporation on n-type Si (Figure 18) with thickness values of *t* = 10, 15, and 25 nm.

**Figure 19:**
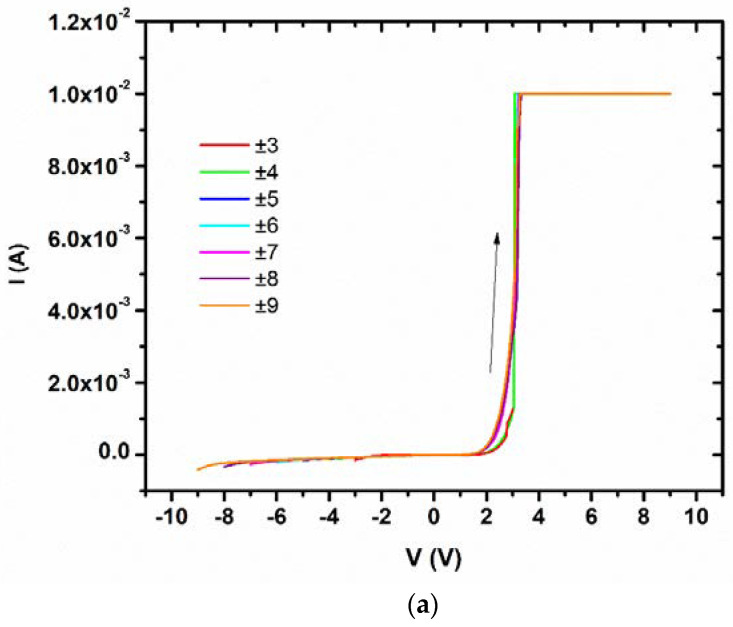

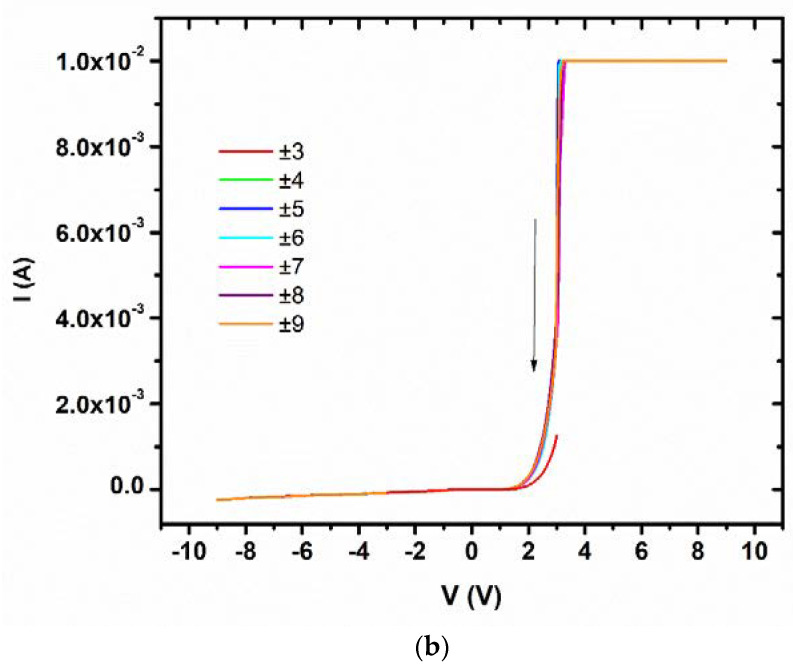
The typical *I*–*V* dependence in the (**a**) forward and (**b**) backward direction of the 10 nm thick MoO_2.96_-based devices in various voltage ranges [77].

Electrical measurements were carried out on all devices, the measurements of a 10 nm thick layer of MoO_3_ with reversible electrically driven MITs being illustrated in Figure 19. Table 1 summarizes the results of all measurements and devices [76].

**Figure 20:**
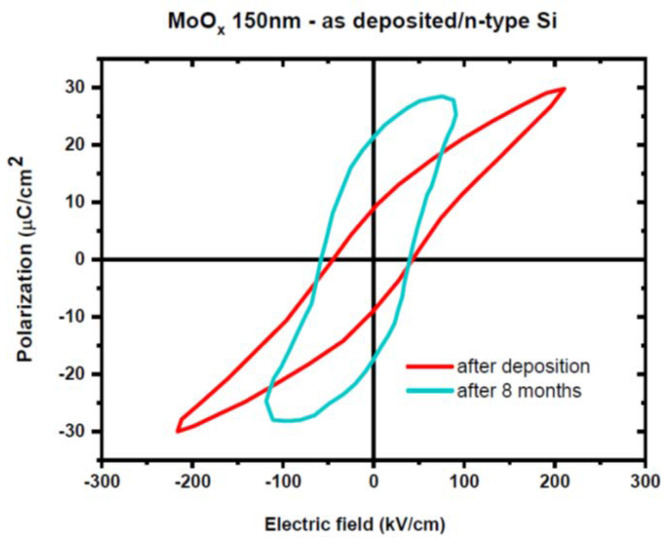
The P-E dependence of MoO_2.96_ thin films, with a thickness of 150 nm, deposited on a Si substrate [77].

The electric field threshold for MIT is 0.034 V/Å and there are differences of six orders of magnitude between the resistivity of the insulator state (27.5 MΩ at −9 V) and that of the metallic state (80 Ω between +5 and +9 V). The ferroelectricity was found for a thicker MoO_3_ of 150 nm as deposited on n-Si (Figure 20); the P-E loops exhibit a coercive field of about 50 kV/cm, a saturation polarization of 30 μC/cm^2^, and a remanent polarization of 10 μC/cm^2^.

## 4. Conclusions

We have presented the electric-field-induced reversible Mott metal-insulator materials and their main applications in nanoelectronics. The foremost obstacle against the development of these applications in nanoelectronics is the deposition of Mott materials, which is a painstaking task. VO_2_ and NbO_2_ have been known for decades as Mott materials, but their growth as MIT materials is still a significant challenge. Therefore, very often we find contradictory results in the literature. This is the main reason why Mott switches have not yet gained widespread used in CMOS and RF microelectronics, and Mott transistors are in their infancy. A promising way forward is to fabricate new Mott structures by creating heterostructures between Mott materials and ferroelectrics, such as NiO and MoO_3_, which are relatively easy to grow in a controllable way, and require very low voltages to produce an MIT. Their ferroelectricity enhances the capabilities of Mott materials for memory and neuromorphic applications.

## Figures and Tables

**Table 1:** The physical characteristics of the MoO_2.96_ MIT [77].

MoO_2.96_ Thickness (nm)	Nr. of Measured Devices	Threshold of Phase Transition (V)	Hysteresis Width (V)	*SS* (mV/Decade)
10	18	3	0.1	48.3
15	19	5	0.1	48
25	17	2.5	0.3	48
